# Toxicological profiling and health hazard characterization of pesticides widely used in Tanzania unmarked

**DOI:** 10.1016/j.toxrep.2026.102269

**Published:** 2026-05-05

**Authors:** Jones A. Kapeleka, Aiwerasia Ngowi

**Affiliations:** a Tanzania Plant Health and Pesticides Authority (TPHPA), Arusha, Tanzania; b Department of Environmental and Occupational Health, Muhimbili University of Health and Allied Sciences (MUHAS), Dar-es-Salaam, Tanzania

**Keywords:** Highly Hazardous Pesticides, Pesticide Exposure, Pesticide Toxicity, Human Health, Organophosphates, Carbamates, Pesticide Poisoning

## Abstract

The toxicity of many pesticides to humans and their contribution to various health problems continue to increase rapidly. This study highlights the inherent effects of highly hazardous pesticides (HHPs) used in smallholder agricultural systems. Primary data were collected through interviews, while secondary data came from scientific databases and search engines, including Scopus, PubMed, Web of Science, and Google Scholar. A total of 810 respondents participated in the study. The characterization of health and environmental impacts was based on the active ingredients mostly used, especially those that have been discontinued, withdrawn, or banned in other regions due to their harmfulness. The toxicological profile focused on effects such as neurotoxicity, reproductive toxicity, developmental toxicity, immunotoxicity, genotoxicity, carcinogenicity, and endocrine disruption. Results show that over 150 active ingredients are used in pesticides, some of which are classified as highly hazardous pesticides (HHPs). Key pesticides like lambda-cyhalothrin, glyphosate, 2,4-dimethylamine, mancozeb, chlorpyrifos, profenofos, cypermethrin, carbendazim, paraquat, atrazine, and carbofuran dominate smallholder farming. The widespread use of HHPs in Tanzania, under current conditions, constitutes a significant public health and environmental crisis, suggesting that knowledge does not always translate into safe practices. The combination of inherently hazardous pesticides, inadequate regulatory oversight, and unsafe handling practices creates a perfect storm of exposure. Strengthening laws and regulations around pesticide registration, post-market monitoring, and enforcement is urgently needed to protect human health and the environment.

## Highlights

• There is high use of Highly Hazardous Pesticides (HHPs) in smallholder farming.
• HHPs are used by unknowledgeable farmers within a compromised regulatory framework.
• Pesticide use and a weak regulatory framework contribute to different health pathologies.
• HHPs are a neglected subject of public health crises that demand urgent action.
• Study backs up a growing pressure for withdrawal of HHPs in developing countries.

## Graphical Abstract

**Figure:**
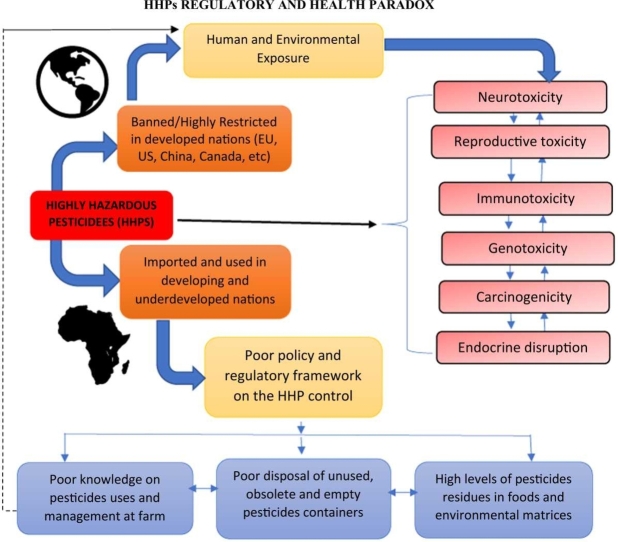

## 1 Introduction

Agriculture remains the backbone of Tanzania’s economy, contributing approximately 25% of foreign exchange earnings, 30% of GDP, 65% of industrial raw materials, 70% of all employment, and 100% of the country’s food needs. [1]. Pesticide use has increased significantly in efforts to manage pest-related crop losses. But this highlights great concerns related to human health, environmental sustainability, and regulatory compliance [2]. This agricultural production is widely dominated by the use of synthetic pesticides [3].

Pesticides are defined by Plant Health Act No. 4 (2020) as a substance, a mixture of substances, or a living organism which includes insecticides, herbicides, fungicides, rodenticides, nematicides, avicides, molluscicides, and antimicrobials intended for preventing, destroying, repelling, or mitigating pests and/or diseases. For decades, the scientific literature has highlighted the toxic effects of pesticides and their role in the development of chronic diseases such as neurological and reproductive disorders, as well as cancers [4], [5]. Exposure to highly hazardous pesticides poses challenges, demonstrating adverse effects on the nervous system and the effects of chronic low-level exposures, such as genotoxicity, carcinogenicity, teratogenicity, sensitization, and neurotoxicity, psychological, immune system, and behavioral dysfunctions, and blood disorders. Pesticides can also cause hormonal imbalance, which can lead to infertility. Long-term exposure to pesticides has been associated with an increased risk of cancer [6], [7].

Exposure to pesticides can cause both acute and chronic health effects. Most cases of acute pesticide poisoning involve either organophosphates or carbamates. They both affect humans by inhibiting acetylcholinesterase, an enzyme essential for proper nervous system function [8], [9]. In Tanzania, like many other developing countries, organophosphates (OPs) and carbamates (CA) constitute the main class of pesticides used [10]. Long-term exposure to some of these pesticides has possible effects, such as cancer, mutations, birth defects, liver and nerve damage, reproductive disorders [11], [12], [13], allergic reactions, and interference with endocrine systems [14]. Unfortunately, health care providers (HCPs) in developing countries face challenges in recognizing, diagnosing, and treating acute pesticide poisoning (APP) due to a lack of training and awareness, leading to underreporting and potentially delayed treatment [15], [16].

The indiscriminate use of pesticides causes severe risks not only in agricultural fields, but also to workers in their manufacturing processes and individual use in homes and institutions. They can enter organism cells, bioaccumulate in food chains, and thus impact human health, depending on their chemical properties [17]. Epidemiological studies have shown that pesticide exposures affected women's production, resulting in menstrual cycle disorders, early menopause, longer time to pregnancy, polycystic ovary syndrome, primary ovarian insufficiency, infertility, and implantation failure [18].

Generally, people exposed for a prolonged or high-intensity time period, particularly agricultural workers, are more likely to experience long-term health effects [19]. In this regard, developing countries bear a disproportionate burden of pesticide-related deaths despite using far fewer pesticides. The World Health Organization (WHO) reported that although Africa uses only 25% of the pesticides produced worldwide, it accounts for 99% of the deaths [20].

Tanzania is experiencing an increasing trend in pesticide use, with WHO Class II hazard-classified pesticides dominating smallholder vegetable production. Smuggled or adulterated pesticides, coupled with easy accessibility, increase poisoning risks. [21]. It is estimated that 81% of pesticides are used in the livestock and agricultural sectors [22]. On the other hand, Tanzania is among many developing countries that still use highly hazardous pesticides (HHPs), which are banned in other nations. Moreover, farmers often lack protective gear, training, and proper storage facilities, leading to high exposure [23].

The immediate effects of pesticide exposure could be through oral, respiratory, or skin exposure, manifested in symptoms like stinging of the eyes and skin, headache, throat, nose, and skin itching, abdominal pain, diarrhea, nausea, blurred vision, and vomiting. Pesticides' chronic side effects can be fatal and take years to manifest in neurological system defects, different types of cancers, reproductive hormones, and respiratory disorders [6], [17], [24]. In addition to the effects of chronic low-level exposures, such as genotoxicity, carcinogenicity, teratogenicity, sensitization, and neurotoxicity, the intricate interactions of the various components of pesticide formulations demand thorough toxicological assessments to manage human health risks effectively [6].

Given the nonspecific nature of pesticide-related diseases, the contribution of pesticides to these noncommunicable diseases remains invisible in the absence of studies linking these pathologies to pesticide exposure. The study aimed to conduct toxicological profiling and assess the potential health and environmental impacts of these substances based on scientific evidence.

The significance of findings is to inform actionable preventive measures for Tanzania and other developing countries disproportionately affected by the adverse effects of hazardous pesticides, and to provide a leeway to strengthen the legal and regulatory framework for post-market surveillance and enforcement to protect human health and the environment.

## 2 Materials and methods

### 2.1 Study area

The present study aims to conduct toxicological profiling and assess its potential health and environmental impacts based on scientific evidence in Tanzania. The study was therefore undertaken in the highest agricultural production zones covering a broad range of production systems in Tanzania [10]. These areas included the northern zone (Arusha and Manyara), the Southern highlands (Iringa and Mbeya), the Lake zone (Mwanza and Shinyanga), the Coastal zone (Dar es Salaam, Mtwara, and Morogoro), and the Western zone (Simiyu).

### 2.2 Sample size and sample selection

The survey primarily involved agricultural producers, pesticide distributors, and dealers. A sample of 810 respondents (704 farmers and 106 retailers) was obtained through a convenience sampling technique [25].

### 2.3 Data sources and collection procedures

Primary data was collected through in-depth field surveys and interviews. Questionnaires containing closed and open-ended questions were translated into Kiswahili, an official language in Tanzania, and administered to farmers, agro-dealers, and pesticide importers through face-to-face interviews by trained interviewers using Kobo Collect [26]. Secondary data sources included the Tanzania Plant Health and Pesticides Authority (TPHPA) as the regulatory authority on registrations, management, and control, and the Ministry of Agriculture reports. For regulatory information outside Tanzania, the databases of the European Food Safety Authority (EFSA), the European Chemicals Agency (ECHA), or the Environmental Protection Agency (EPA) were consulted. Data were also collected from various bibliographic databases and search engines, including PubMed, Web of Science, and Google Scholar databases, using the keywords "Name of the active substance" and "toxic effect". Characterization of health and environmental impacts was based on registered active ingredients, particularly those discontinued, withdrawn, or banned from other regions, including the European market, the US, China, and Canada, based on their harmfulness [27].

### 2.4 Data processing and analysis

Data were processed and analyzed using the statistical program SPSS version 22 (SPSS Inc., Chicago, IL, USA). Descriptive statistics, such as frequencies, were used to describe the data. For each selected active substance, a detailed analysis was done to ascertain what is known about its toxicity based on the scientific literature and available regulatory dossiers from different regulatory bodies. With the aid of a color code, toxicological information of each substance was organized according to the type of toxic effects sought: neurotoxicity, reproductive toxicity, endocrine disruption, genotoxicity, and carcinogenicity, and their European regulation, and according to the type of approach in vitro, in vivo, or inhuman.

### 2.5 Research clearance

Research clearance for the study was granted by the Commission for Science and Technology (COSTECH) after a formal application. Participants were included in the study after their free and informed consent. The information collected was kept confidential within the research team, with survey data being anonymized.

## 3 Results

### 3.1 Demographic characteristics of respondents

A total of 704 farmers were included in the study, the majority of whom were male (79.5%, 95% CI: 76.4–82.5), while females constituted 20.5% (95% CI: 17.5–23.6). With respect to marital status, most respondents were married (87.9%, 95% CI: 85.3–90.2), followed by single individuals (11.1%, 95% CI: 8.9–13.6). Regarding occupation, farming was the dominant activity, reported by 85.8% (95% CI: 83.0–88.3) of respondents. Nearly all respondents were individual pesticide users (99.7%, 95% CI: 99.0–100.0), with negligible representation from crop boards and the Ministry of Agriculture (0.1% each). (Table 1).

**Table 1:** Demographic characteristics of farmers.

**Variable**	**Category**	**n**	**Total**	**Percent**	**CI: Lower**	**CI: Upper**	**Percent:CI**
Sex	Male	560	704	79.5	76.4	82.5	79.5% (76.4–82.5)
	Female	144	704	20.5	17.5	23.6	20.5% (17.5–23.6)
Marital status	Married	619	704	87.9	85.3	90.2	87.9% (85.3–90.2)
	Single	78	704	11.1	8.9	13.6	11.1% (8.9–13.6)
	Widowed	4	704	0.6	0.2	1.4	0.6% (0.2–1.4)
	Divorced	3	704	0.4	0.1	1.2	0.4% (0.1–1.2)
Main Occupation	Farmer	599	698	85.8	83	88.3	85.8% (83–88.3)
	Business	45	698	6.4	4.7	8.5	6.4% (4.7–8.5)
	Livestock keeper	29	698	4.2	2.8	5.9	4.2% (2.8–5.9)
	Technician	16	698	2.3	1.3	3.7	2.3% (1.3–3.7)
	Civil servant	9	698	1.3	0.6	2.4	1.3% (0.6–2.4)
Categories of pesticide users	Individual farmer	695	697	99.7	99	100	99.7% (99–100)
	Crop Board	1	697	0.1	0	0.8	0.1% (0–0.8)
	Ministry of Agriculture	1	697	0.1	0	0.8	0.1% (0–0.8)

### 3.2 Registration status and management of pesticide empty containers

Most farmers reported keeping leftover pesticides (76.0%, 95% CI: 72.5–79.2). A smaller proportion engaged in unsafe practices such as re-spraying crops (10.1%, 95% CI: 7.9–12.7) or discarding residues on farms (5.0%, 95% CI: 3.5–7.0). Burial (2.8%, 95% CI: 1.6–4.3) and disposal in water (0.3%, 95% CI: 0.0–1.1) were less common. The most frequent disposal method was discarding in nature (36.6%, 95% CI: 32.9–40.5), followed by burial (31.3%, 95% CI: 27.8–35.0) and open burning (26.1%, 95% CI: 22.8–29.6). Only 0.2% (95% CI: 0.0–0.8) returned containers to suppliers. A high proportion of pesticides used were reported as registered with TPHPA (96.9%, 95% CI: 94.8–98.3), most had labels in both Kiswahili and English (97.7%, 95% CI: 95.8–98.9) (Table 2).

**Table 2:** Pesticide registration and management of pesticide empty containers.

**Variable**	**Category**	**n**	**Total**	**Percent**	**CI:Lower**	**CI:Upper**	**Percent:CI**
What do you do with leftover products?	Kept	497	654	76	72.5	79.2	76% (72.5–79.2)
	Re-spray crops-Double passage	66	654	10.1	7.9	12.7	10.1% (7.9–12.7)
	No leftover	38	654	5.8	4.1	7.9	5.8% (4.1–7.9)
	Discarded on the farm	33	654	5	3.5	7	5% (3.5–7)
	Buried	18	654	2.8	1.6	4.3	2.8% (1.6–4.3)
	Discarded in water	2	654	0.3	0	1.1	0.3% (0–1.1)
What do you do with empty pesticide packaging/containers after use?	Discarded in nature	240	655	36.6	32.9	40.5	36.6% (32.9–40.5)
	Buried	205	655	31.3	27.8	35	31.3% (27.8–35)
	Burnt in an open fire	171	655	26.1	22.8	29.6	26.1% (22.8–29.6)
	Domestic use	38	655	5.8	4.1	7.9	5.8% (4.1–7.9)
	Returned to suppliers	1	655	0.2	0	0.8	0.2% (0–0.8)
Pesticide Registered by TPHPA	YES	437	451	96.9	94.8	98.3	96.9% (94.8–98.3)
	NO	14	451	3.1	1.7	5.2	3.1% (1.7–5.2)
Label translated into Kiswahili and/or English	Kiswahili and English	424	434	97.7	95.8	98.9	97.7% (95.8–98.9)
	English Only	10	434	2.3	1.1	4.2	2.3% (1.1–4.2)

### 3.3 Pesticide use and handling practices

Almost all farmers reported experiencing crop pest problems (99.6%, 95% CI: 98.7–99.9) and using pesticides (96.7%, 95% CI: 95.0–97.9), indicating a near-universal reliance on chemical pest control. The primary source of pesticides was retail shops (82.0%, 95% CI: 78.8–84.8), followed by crop boards (6.4%, 95% CI: 4.7–8.6) and cooperatives (5.2%, 95% CI: 3.6–7.2). Informal sources such as open markets and vendors contributed minimally. Farmers mainly relied on pesticide retailers (37.3%) and extension services (31.9%) for information. Very few obtained guidance from regulatory authorities such as TPHPA (0.2%). On the other hand, a high proportion of farmers reported reading pesticide labels (90.8%, 95% CI: 87.4–93.4), suggesting awareness of instructions. However, application methods were almost exclusively limited to knapsack sprayers (97.5%, 95% CI: 95.9–98.6). Re-entry intervals varied, with 33.5% (95% CI: ∼29–38) waiting 72 h, while 9.9% (95% CI: ∼7–13) re-entered fields immediately after spraying (Table 3).

**Table 3:** Pesticide use and handling practices.

**Variable**	**Category**	**n**	**Total**	**Percent**	**CI: Lower**	**CI: Upper**	**Percent:CI**
Have problems with crop pests on your farm	Yes	690	693	99.6	98.7	99.9	99.6% (98.7–99.9)
	No	3	693	0.4	0.1	1.3	0.4% (0.1–1.3)
Do you use pesticides for your agricultural activities?	Yes	665	688	96.7	95	97.9	96.7% (95–97.9)
	No	23	688	3.3	2.1	5	3.3% (2.1–5)
How do you get the pesticides you use?	Pesticides retail shop	536	654	82	78.8	84.8	82% (78.8–84.8)
	Crop board	42	654	6.4	4.7	8.6	6.4% (4.7–8.6)
	Cooperative Society (AMCOS)	34	654	5.2	3.6	7.2	5.2% (3.6–7.2)
	Pesticides company	14	654	2.1	1.2	3.6	2.1% (1.2–3.6)
	At the open market	10	654	1.5	0.7	2.8	1.5% (0.7–2.8)
	From the government Extension	9	654	1.4	0.6	2.6	1.4% (0.6–2.6)
	From NGOs	6	654	0.9	0.3	2	0.9% (0.3–2)
	Ambulant merchant (Vendors)	2	654	0.3	0	1.1	0.3% (0–1.1)
	Through tendering	1	654	0.2	0	0.8	0.2% (0–0.8)
Where do you get information on how to use pesticides?	Pesticide retailer	241	646	37.3	33.6	41.2	37.3% (33.6–41.2)
	Extension services	206	646	31.9	28.3	35.6	31.9% (28.3–35.6)
	Have access to any information source	100	646	15.5	12.8	18.5	15.5% (12.8–18.5)
	Technical Service for Agriculture	43	646	6.7	4.9	8.9	6.7% (4.9–8.9)
	A friend	38	646	5.9	4.2	8	5.9% (4.2–8)
	A family member	9	646	1.4	0.6	2.6	1.4% (0.6–2.6)
	Association of pesticide vendors	8	646	1.2	0.5	2.4	1.2% (0.5–2.4)
	TPHPA	1	646	0.2	0	0.9	0.2% (0–0.9)
Do you read pesticide labels before using pesticide products	Yes	335	369	90.8	87.4	93.5	90.8% (87.4–93.5)
	No	34	369	9.2	6.5	12.6	9.2% (6.5–12.6)
What equipment do you use to apply these pesticides	Knapsack-Back Sprayers	624	640	97.5	96	98.6	97.5% (96–98.6)
	Motorized sprayer	16	640	2.5	1.4	4	2.5% (1.4–4)
Re-entry to the newly treated parts of your field	72 h	203	606	33.5	29.7	37.4	33.5% (29.7–37.4)
	24 h	182	606	30	26.4	33.9	30% (26.4–33.9)
	48 h	161	606	26.6	23.1	30.3	26.6% (23.1–30.3)
	Immediately after the spray	60	606	9.9	7.6	12.6	9.9% (7.6–12.6)

### 3.4 Formulations of widely used pesticides

**Fig. 1:**
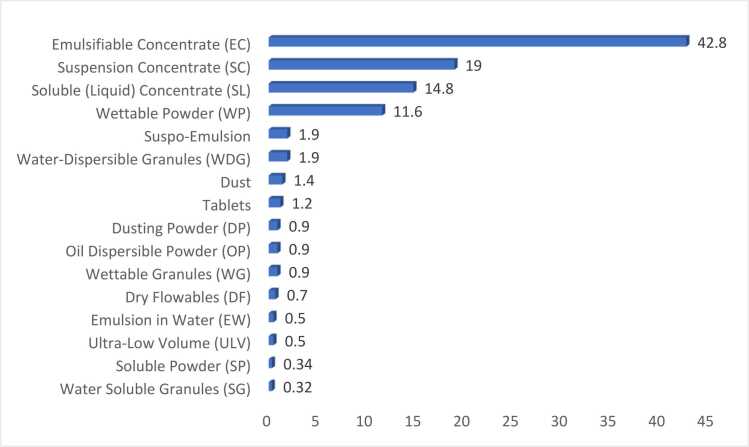
Formulations of widely used pesticides.

Emulsifiable concentrate (EC) (42.8%), Suspension concentrate (SC) (19.0%), Soluble (liquid) concentrate (SL) (14.8%), and Wettable powder (WP) (11.6%) constitute the most common pesticide formulations. A very small proportion of ULV formulations, which demand specialized sprayers and spraying equipment, was also reported (Fig. 1).

### 3.5 WHO toxicity class of pesticides sold by pesticide retailers

**Fig. 2:**
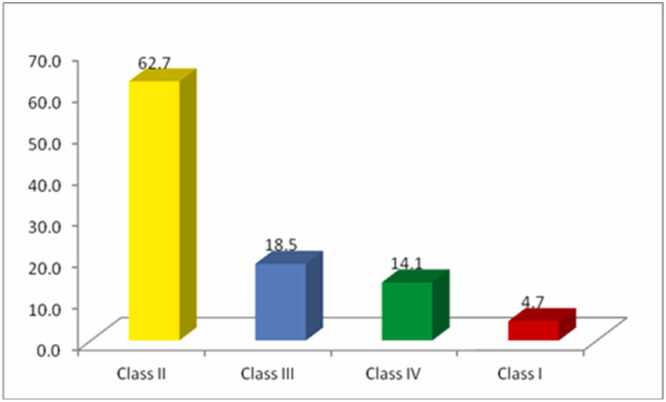
WHO toxicity Class of pesticides sold by pesticide retailers.

All pesticide toxicity classes, ranging from Class I (extremely toxic) to Class IV (slightly toxic), were found to be sold and used by farmers. Most pesticides (62.7%) fall under Class II of the toxicity under the WHO classification (Highly toxic), with a small proportion of extremely toxic Class I (Fig. 2).

### 3.6 Common active ingredients used by farmers

Local pesticide shops supply a wide range of pesticides. Over 286 different pesticide trade names dominate the pesticide market, with over 150 active ingredients available to farmers. The highly used active ingredients were Lambda-cyhalothrin 50 g/l (5.7%), Glyphosate 480 g/l (5.4%), Hexaconazole 50 g/l (3.4%), 2,4-Dimethylamine salt 720 g/l (3.2%), and Dimethoate 400 g/l, mixed formulation of Metalaxyl 80 g/kg +Mancozeb 640 g/kg (2.9%) respectively, Sulphur 800 g/kg (2.5%), Chlorpyrifos 480 g/l and Profenofos 500 g/l (2.3%) respectively. Others include Cypermethrin, Carbaryl, carbendazim, triadimefon, triadimenol, paraquat, atrazine, Chlorothalonil, and carbofuran. Most of these active ingredients have not been approved for use in the EU (Table 4).

**Table 4:** Toxicological profile of pesticides commonly used in Tanzania.

**SN**	**Chemical name**	**Acute toxicity (Oral LD₅₀ (rat): mg/kg**	**Chronic toxicity**	**ADI/ARfD (mg/kg bw/day)**	**Mechanism of action**	**Ecotoxicological risk**	**EU Approval**
1	2,4-Dimethylamine	425–764	Neurotoxicity/ Delayed neurotoxicity, Reproductive toxicity	0.02	Reproduction/development effects	Moderately toxic to birds and most aquatic species, as well as honeybees and earthworms.	Approved
2	Atrazine	1869	Circulatory collapse, gastric bleeding, and renal failure. May disturb testosterone metabolism, androgen inhibition, and weak estrogenic effect	0.02	Endocrine disruptor	Moderately toxic to most aquatic life, earthworms, and honeybees,	Not approved
3	Carbaryl	614	Reproduction/development effects	0.0075	Acetyl cholinesterase inhibitor, Endocrine disruptor	Highly toxic to mammals, moderately toxic to birds, fish, and algae.	Not approved
4	Carbendazim	> 10000	Reproduction/development effects	0.02	Reproduction/developmental toxicant	Moderately toxic to honeybees and most aquatic organisms, highly toxic to earthworms,	Not approved
5	Carbofuran	7	Has a high mammalian toxicity, endocrine disruptor, and is a probable reproductive/developmental intoxicant	0.00015	Acetyl cholinesterase inhibitor	Highly toxic to birds and honeybees, moderate to high toxicity to most aquatic organisms	Not approved
6	Chlorothalonil	> 5000	Probable human carcinogen	0.015	Endocrine disruptor	Moderately toxic to birds, honeybees, and earthworms, more toxic to aquatic organisms	Not approved
7	Chlorpyrifos	135	Highly toxic to mammals, classified as a reproductive toxicant and a neurotoxicant	0.003	Acetyl cholinesterase inhibitor	Very toxic to bees, fish, and birds	Not approved
8	Cypermethrin	287	Possible human carcinogen, Estrogenic effect, and Possible liver & kidney toxicant	0.005	Endocrine disruptor	Highly toxic to most aquatic species and honeybees	Approved
9	Dimethoate	245	Disruption of thyroid hormones' action, Possible liver toxicant, and neurotoxic	None allocated	Acetyl cholinesterase inhibitor	Highly toxic to birds and honeybees	Not approved
10	Glyphosate	> 2000	Possible bladder and liver toxicant, may cause serious eye damage, gastrointestinal disturbances, and Disruption of aromatase activity	0.5	Neurotoxic effects affect brain development, with potential consequences on behavior, and the development of autism disorders	Moderately toxic to birds, most aquatic organisms, earthworms, and honeybees	Approved
11	Hexaconazole	2189	Possible human carcinogen, Inhibition of aromatase activity, decrease in estrogen production	0.005	Endocrine disruptor	Moderately toxic to birds, fish, aquatic invertebrates, algae, and earthworms,	Not approved
12	Lambda-cyhalothrin	56	Harmful if swallowed, inhaled, or in contact with skin. Possible immune system and thyroid toxicant in susceptible individuals	0.0025	Endocrine disruptor, estrogenic effects, reproductive and developmental toxicity, sexual dysfunction	Highly toxic to fish, aquatic invertebrates, and honeybees, moderately toxic to earthworms	Approved
13	Mancozeb	> 5000	Possible thyroid toxicant, may cause ovarian hypertrophy, and probable human carcinogen	0.023	Reproduction/development effects	Highly toxic to fish and aquatic invertebrates, and moderately toxic to birds and earthworms	Not approved
14	Paraquat	110	Potential liver, kidney, stomach, intestine, and respiratory system toxicant	0.004	Respiratory tract, skin, and eye irritant	Highly toxic to mammals and birds	Not approved
15	Permethrin	> 430	Moderately toxic to humans, is an irritant and may be a CNS toxicant.	None allocated	Reproduction/development effects, Endocrine disruptor, and Neurotoxicant	Highly toxic to most aquatic species and honeybees,	Not approved
16	Profenofos	358	Acetyl cholinesterase inhibitor and neurotoxicant	0.03	Acetyl cholinesterase inhibitor and neurotoxicant	Highly toxic to honeybees and birds	Not approved
17	Triadimefon	300	Liver and thyroid toxicant, Estrogenic	0.03	Neurotoxicity, liver damage, and developmental abnormalities induce oxidative stress	Highly toxic to aquatic organisms	Not approved

## 4 Discussion

The use of pesticides has been scientifically proven to hurt both human and environmental health. Generally, organophosphorus insecticides were the most potent and widespread inhibitors [28]. The study population is highly homogeneous, dominated by males, married, and farming individuals, indicating that pesticide exposure risks are concentrated within traditional smallholder farming systems. Based on scientific evidence, farming communities in Tanzania, along with those in other developing countries, are at risk due to an inadequate regulatory system for pesticide control. This toxicological profile of pesticides reveals a critical public health and environmental challenge in Tanzania.

The pesticides most widely used in the country predominantly meet the criteria for classification as Highly Hazardous Pesticides (HHPs). While pesticide registration and labeling appear well implemented, post-use management practices are poor, with widespread environmentally unsafe disposal methods. This indicates a gap between regulatory compliance and field-level practices. Their use occurs within a system characterized by an inefficient regulatory framework [23], unsafe farming practices and a lack of local data on residue exposure place farmers, consumers, and ecosystems at significant risk. Scientific studies on the use of these pesticides have derived a significant association between chronic pesticide exposure and non-communicable diseases, including cancer, neurological disorders, and endocrine disruptions.

Exposure to organophosphates, with their large distribution and lipophilic characteristics, quickly spreads into the liver, kidneys, and adipose tissue, providing a defense against metabolism. Similar to Ops, Carbamate pesticides target the nervous system, inhibiting cholinesterases enzyme activity, causing symptoms like breathing difficulties, muscle weakness, twitching, and, in severe cases, respiratory paralysis, seizures, and coma [19]. This study highlights the inherent toxicity of these pesticides, including compounds known to be neurotoxic, endocrine-disrupting, reprotoxic, and potentially carcinogenic. [5], [29], [30]. The documented pesticides pose a range of serious health threats, which can be grouped into primary effect categories:

*Neurodevelopmental and Neurological Toxins*: A major concern is the widespread use of insecticides linked to permanent neurological damage, particularly in children. Chlorpyrifos and other organophosphates like profenofos, Carbofuran, Dimethoate (banned in Europe) are acetylcholinesterase inhibitors, while pyrethroids like cypermethrin and lambda-cyhalothrin disrupt nerve impulse transmission. Exposure to lambda-cyhalothrin during early gestation may pose a threat to pregnancy [31]. Epidemiological evidence is unequivocal: prenatal and childhood exposure to chlorpyrifos is associated with reduced IQ, developmental delays, and ADHD [5], [32]. Studies have shown that paraquat causes oxidative stress and mitochondrial dysfunction, contributing to nerve cell death in Parkinson’s disease [29]. EPA banned the use of chlorpyrifos in food crops because of the correlation between exposure to this pesticide and neurological damage, especially in children [33]. Furthermore, chlorpyrifos has been linked to the deformation of brain morphology in young children [34].

Permethrin is neurotoxic. Its neurotoxicity is associated with congenital deficits and neurobehavioral disturbances [35] subjecting Tanzanian children at risk [19], since farms and homes are often in proximity, exposing children directly. Furthermore, 2,4-D, one of the most used herbicides in Tanzania, induces neurotoxicity with neurobehavioral deficits [36].

Profenofos had been reported to be neurotoxic. It affects the cholinergic and non-cholinergic neurotransmission systems [37] and neurobehavioral dissonance in exposed individuals [38].

*Endocrine Disruptors and Reproductive Toxicants*: Many of the identified pesticides interfere with the hormonal system. Neurodegenerative illnesses, including Parkinson's and Alzheimer's, have been linked to this oxidative damage as a result of exposure to OPs and CA [19]. The fungicide mancozeb and the herbicides atrazine and 2,4-D have been shown to disrupt menstrual cycles, reduce testosterone levels, and cause sperm abnormalities [39], [40], [41]. Endocrine-disrupting chemicals (EDCs) can mimic or block natural hormones by binding to receptors, such as estrogen, progesterone, aryl hydrocarbon, or thyroid-stimulating receptors, disrupting hormone synthesis, secretion, and metabolism [42]. Mancozeb induces longer menstrual cycles in exposed women and developmental injury in pre-implantation embryos, and is also linked to the development of Parkinson’s disease [43]. Chemical toxicants from OPs and CA can disrupt the endocrine system, affecting the transport, synthesis, binding, secretion, and elimination of natural hormones. These internal body mechanisms are vital for homeostasis, reproduction, development, and behavior [19], [44]. In recent years, fertility rates have declined significantly, while adverse reproductive outcomes have increased, partly due to pesticide exposure. EDCs have shown the ability to initiate carcinogenic changes in breast tissue or accelerate cancer progression [42]. Women exposed to pesticides, therefore, face higher risks of reproductive problems [7]. However, insecticides are among the substances that may lower the quality of the semen produced by exposed workers [24]. This signifies the increased incidence of reproductive disorders in the country [7], [18]. Lambda-cyhalothrin and cypermethrin exhibit estrogenic effects, potentially influencing breast cancer cell proliferation. This endocrine-disrupting action, often occurring at low doses, threatens reproductive health across generations. Chronic exposure to 2,4-D results in impaired neurobehavioral function and induces reprotoxicity [36] through decreased testosterone and an increased rate of sperm abnormalities. Exposure to dimethoate and carbofuran causes disruption of thyroid hormones' action, liver toxicity, and reproductive/developmental disorders [45]. Pyrethroids, among the widely used pesticide groups in Tanzania, harm male reproductive systems through DNA damage and rise in spermatozoa with malformed heads, followed by degeneration and mortality [24].

Glyphosate is widely used as a herbicide in Tanzania. It has been described as an endocrine disruptor and reproductive pesticide [46]. Furthermore, exposure to glyphosate is linked to the development of Parkinson’s disease [47]. Atrazine is another herbicide widely used in Tanzania. Toxicologically, it is associated with an increased prevalence of short stature for gestational age [48]. Regarding the gender-based farming system in Tanzania, women are at high risk of exposure as they are highly involved in weeding and/or harvesting the sprayed field. Lambda-cyhalothrin has been found to exert reprotoxicity and developmental toxicity, causing sexual dysfunction [49] and a threat to pregnancy [31]. In this regard, infants and children are more vulnerable to the adverse effects of these pesticides due to their developing bodies and the potential for both acute and chronic exposure consequences [50].

*Carcinogenic and Genotoxic Potential:* Several pesticides raise concerns regarding cancer risk. OPs are known for their high toxicity, affecting the nervous system. This can lead to severe health issues, including cancer and damage to multiple organs [50]. Several pesticides found to be used by farmers are carcinogens. The IARC classifies glyphosate as a "probable human carcinogen" (Group 2 A) and 2,4-D as "possibly carcinogenic" (Group 2B), with meta-analyses suggesting links to non-Hodgkin lymphoma [51], [52], [53], [54], [55]. Chronic low-level exposure to glyphosate has been linked to epigenetic changes, DNA damage, oxidative stress, and immune disruption, which contribute to diseases such as cancer and immune disorders [56], leading to gene mutations that could be inherited from father to child [24]. Chlorothalonil is classified as a "probable human carcinogen" by the EPA, and carbaryl is suspected of causing cancer. While epidemiological data can be complex, the genotoxic potential of these compounds—evidenced by DNA damage in human cells from glyphosate, chlorothalonil, and paraquat provides a mechanistic basis for concern [57]. Several epidemiological studies show a positive association of these pesticides with the risk of non-Hodgkin lymphoma [51], [52], [53]. Paraquat had also been found to induce genotoxicity in human liver HepG2 cells and human lymphocytes via frequency of chromosome aberrations and frequency of micronuclei [57], while cypermethrin has been linked to exerting genotoxicity [58]. Paraquat had been linked to fatal human pathologies, was found to reduce kidney function in agricultural workers [59]. It had been linked to causing renal failure [60]. Paraquat dichloride had been the main cause of many fatalities due to accidental or voluntary ingestion [43]. Suicide attempts by paraquat ingestion lead to kidney damage [61].

Likewise, a study in the United States showed a positive association between end-stage renal disease in wives of pesticide applicators and husbands' use of paraquat [62]. Parkinson's disease was also positively associated with paraquat use among agricultural pesticide users, particularly in younger subjects and/or when exposure occurs at a younger age [63]. On the other hand, Lambda-cyhalothrin increased the number of structural chromosomal aberrations and the frequency of micronucleated erythrocytes [64], causing significant DNA damage.

Exposure to chlorpyrifos increases the likelihood of breast cancer in women [65] while Cypermethrin induces genotoxicity in human peripheral blood lymphocytes [66], [67] and in human hepatic cancer cells, HepG2 [68]. Cypermethrin can also induce transplacental genotoxicity in [58] and significant genetic damage in the tissues of the progeny when exposed during gestation [69].

*Specific Organ Toxicity*: Paraquat is notorious for causing acute, often fatal, lung and renal failure upon ingestion and is strongly linked to Parkinson's disease [70]. Exposure to mancozeb and its metabolite has a significant impact on thyroid function via hypothyroidism [71]. Prenatal exposure to pesticide mixtures containing mancozeb shows adverse developmental effects, including disruption of brain development [72]. Studies on mancozeb also illustrate the correlation between oxidative stress and liver damage [6]. On the other hand, the use of paraquat is highly linked to suicide attempts due to its acute kidney damage [61].

This study reports a huge number of pesticide formulations and active ingredients used by farmers far beyond the numbers reported elsewhere in Africa [73]. The study also identified the vulnerable context of pesticide use. Despite high pesticide use and reported label reading, practical safety behaviors are inconsistent, particularly regarding re-entry intervals and reliance on informal information sources. The regulatory system hardly protects a largely uninformed farming population, who, due to a lack of access to personal protective equipment (PPE) and training, have easy access to hazardous pesticides and handle these toxic substances with minimal protection [16]. Practices such as storing leftovers in open containers in living quarters and re-spraying leftovers drastically increase the risk of human exposure and environmental contamination [21].

The risk is not solely from the active ingredients. Pesticide formulations (e.g., emulsifiable concentrates - EC) have been reported to be more toxic than their pure active ingredients [74]. Furthermore, the lack of available PPE means farmers are applying these potent formulated products with direct dermal and inhalation exposure, a risk factor not captured in dietary risk assessments. Chronic exposure to pesticides presents significant health risks. Individuals in high-exposure environments, such as farming, are prone to health effects, as evidence indicates strong associations with several long-term health conditions [29]

A striking finding of this profile is the profound regulatory dissonance. Tanzania authorizes numerous pesticides that have been banned or phased out in the European Union and other countries due to unacceptable risks. This includes: Chlorpyrifos: Banned in the EU and for food crops in the U.S. due to brain damage in children. Paraquat: Banned in the EU and Switzerland due to its high acute toxicity and link to Parkinson's. Atrazine: Banned in the EU due to persistent groundwater contamination. Chlorothalonil: Banned in the EU due to its carcinogenicity. Carbaryl and Profenofos: Banned in the EU. This situation creates a double standard where the Tanzanian population and its environment are exposed to risks deemed unacceptable for other regions. This underscores the urgent need for Tanzania to reassess its pesticide authorization process based on the precautionary principle and up-to-date independent science, rather than relying on industry-sponsored data.

The strength of this study is that it shows the number of individual studies performed with different groups of agricultural workers, epidemiological studies, and in vivo, in vitro, and in human studies, all depicting the health effects of exposure to pesticides, mostly those widely used in Tanzania. However, a major shortcoming is the lack of adequate matching data regarding pesticide exposure in the local context (Africa), a lack of chemical exposure measurements across primary effect categories.

## 5 Conclusion

The toxicological evidence of inherent health and environmental risks of the pesticides used is clear and alarming. The widespread use of HHPs in Tanzania, under current conditions, constitutes a significant public health and environmental crisis, most of which are either gradually removed or banned for use in the EU, the US, and other countries. This suggests that knowledge does not always translate into safe practices. Mancozeb, Chlorpyrifos, Profenofos, Lambda cyhalothrin, Permethrin, Atrazine, and glyphosate, among many other pesticides used by farmers in Tanzania, exert high levels of toxicity.

The combination of inherently hazardous pesticides, inadequate regulatory oversight, and unsafe handling practices creates a perfect storm of exposure. Strengthening laws and regulations around pesticide registration, post-market monitoring, and enforcement is urgently needed to protect human health and the environment.

### 5.1 Directions for future research, practice, and policy

Deliberate efforts are required in the regulatory framework to derive strategies that prevent farmers’ easy access to highly toxic pesticides and eventual exposure. Future research should focus on addressing the increasing incidences of non-communicable diseases, including breast, prostate, and colorectal cancers, male and female reproductive dysfunctions, and other health problems, such as Parkinson’s disease, which should therefore be reassessed in relation to pesticide exposure.

Addressing this issue is not merely a regulatory exercise; it is a moral imperative to safeguard the health of Tanzanian farmers, consumers, and children for generations to come. It is fundamental to promote a regulatory framework in pesticide management and control that encourages agricultural practices that are less dependent on chemical pesticides. More efforts and resources should be invested in research that explores long-term impacts and mitigation strategies of adverse effects, both on humans and the environment, and alternatives to chemical pesticides. TPHPA should reinstitute a strong pesticide research arm to foster pesticide research in the country.

We therefore recommend the following actions:

1. Regulatory Review: Review of registered pesticides against HHP criteria, prioritizing the phase-out of those banned elsewhere (e.g., chlorpyrifos, paraquat, atrazine).
2. Investment in Alternatives: Promote and invest in research and adoption of Integrated Pest Management (IPM) and agroecological methods to reduce dependency on chemical pesticides.
3. Farmer Training and Support: Launch nationwide training programs on safe handling, proper application, and storage of pesticides, coupled with subsidies for appropriate PPE.
4. Local Monitoring: Establish a robust national system for monitoring pesticide residues in food, water, and soil to generate local exposure data and inform risk assessments.
5. Policy Reform: Strengthen the legal and regulatory framework for pesticide registration, post-market surveillance, and enforcement to protect human health and the environment.
6. Declare pesticide exposure and health effects as a “Neglected Public Concern” to gain political will and support.

## CRediT authorship contribution statement

**Jones A. Kapeleka:** Writing – original draft, Visualization, Methodology, Investigation, Formal analysis, Data curation, Conceptualization. **Aiwerasia Ngowi:** Writing – review & editing, Validation, Supervision, Project administration, Investigation, Funding acquisition.

## Declaration of Competing Interest

The authors declare the following financial interests/personal relationships which may be considered as potential competing interests. Jones A. Kapeleka reports financial support was provided by The France Embassy in Tanzania. If there are other authors, they declare that they have no known competing financial interests or personal relationships that could have appeared to influence the work reported in this paper.

## Data Availability

The authors are unable or have chosen not to specify which data has been used.
